# Preparation, Properties, and Microbial Impact of Tungsten (VI) Oxide and Zinc (II) Oxide Nanoparticles Enriched Polyethylene Sebacate Nanocomposites

**DOI:** 10.3390/polym13050718

**Published:** 2021-02-26

**Authors:** Amr S. Ismail, Salah M. Tawfik, Amr H. Mady, Yong-Ill Lee

**Affiliations:** 1 Petrochemicals Department, Egyptian Petroleum Research Institute (EPRI), Nasr City 11727, Cairo, Egypt; salahtwfk85@yahoo.com (S.M.T.); amr_mady2005@yahoo.com (A.H.M.); 2 Department of Chemistry, Changwon National University (CNU), Changwon 51140, Korea

**Keywords:** polyester, metal oxide, nanocomposites, microbial activity, nanotechnology

## Abstract

Nanoparticles of tungsten oxide (WO_3_) and zinc oxide (ZnO) enriched polyethylene sebacate (PES) nanocomposites were prepared through the coprecipitation process and condensation polymerization reaction. The obtained nano-sized particles of WO_3_ and ZnO, PES, and nanocomposites (WO_3_-PES NC and ZnO-PES NC) were investigated. The average molecular weight of the cured PES was measured by employing the gel permeation chromatography (GPC) technique. Fourier-transform infrared spectroscopy (FTIR) and X-ray diffraction (XRD) spectra assured the formation of the polymeric nanocomposites.WO_3_ and ZnO nanoparticles supposed a condensed porous spherical phase found implanted in the polymer structure, as detected by scanning electron microscopy (SEM) and transmission electron microscopy (TEM) methods. These nano-scale systems achieved an electrical activity based on the conductive nanoparticles embedded matrix as a result of the ion–ion interactions. The microbial influence of the nanocomposites was examined against pathogenic bacteria; *Pseudomonas aeruginosa,* *Escherichia coli, Staphylococcus aureus,* and *Bacillus subtilis*, and Fungi; *Aspergillus niger,* and *Candida* *albicans*. Results exhibited that these nanocomposites have antimicrobial effects from moderate to slightly high on bacteria and high on fungi which was confirmed by a clear zone of inhibition. This study contributes to the design of reasonable composites to be under evaluation for their catalytic effect.

## 1. Introduction

On the ever-accelerating progress of technology towards sustainable development, there are considerable research outputs in respect of the fabrication of eco-amiable nano-sized material systems with formidable characteristics [1]. Recent advances and upcoming technologies are mainly focused on the overall coverage of the principles and methods concerning productions, properties, and implementations of nanomaterials [2,3]. By their distinguished properties, polymer-metal oxide composites have been widely investigated in recent years [4,5,6,7,8]. In this context, convenient processing procedures for fabricating metal oxides on elastic polymeric materials are highlighted. Metal oxides grafted semicrystalline and partially polymeric films such as polydimethylsiloxane (PDMS), polyethylene naphthalate (PEN), polyethylene terephthalate (PET), and polyimide (PI) have been studied [9,10]. Even though, a number of these amorphous polymeric films do not expose all the coveted characteristics regarding their morphology, stability, resistivity under ambient conditions [10]. Otherwise, soft composites based on linear aliphatic polyester (PE) are successfully employed to modify the polymer structure with new categories of strengthening components owing to their characteristics [11,12,13,14,15]. Predominately, positive reinforcement materials in nano-sized scales were embedded in the cast polymer composites to improve their physicochemical and electromechanical properties [16,17,18,19]. Metal oxides of Al, Ti, Zr, and In with appropriate components are utilized in flexible devices based on their crystalline phases [20]. Compared to these oxide materials, nanoflakes, nanosheets, and nanowires of WO_3_ based polymeric composites were employed efficiently in various usages such as sensitive gas sensors [21,22], dye-sensitized solar cells [23], lithium-ion batteries [24], photochromic agents [25], photocatalytic and antibacterial agents [26,27], supercapacitors [28], water splitting [29,30], wastewater treatment [31], dye removal [32], smart windows [33], and anticancer agents [34]. As a result of the properties of zinc oxide, it has appeared a potential interest in the formulation of nano-sized polymeric systems [35]. Newly, the preparation of convenient components containing ZnO nanoparticles provides effective composites for several implementations such as acetone sensing [36], photocatalysis [37,38], photodegradation [39], microbial activity [40], cancer therapy [41], and wound dressing [42], food packaging [43] and water splitting [44]. Furthermore, the enhanced catalytic activity and promoted thermal-catalytic stability of ZnO nanostructures and its based hybrid materials under visible light effects were reported [45,46,47,48]. Consequently, the present research work endeavored to fabricate a polyester matrix composite of polyethylene sebacate enriched with nanocrystalline oxides of tungsten and zinc. These nano-oxides donate free electrons to the polymer matrix based on the difference in oxidation state. The sequential preparation of PES nanocomposites was achieved by condensation and deposition procedures. The composite systems were investigated. As well, the electrical conductivity and microbial performance of the obtained nanocomposites were examined.

## 2. Experimental

### 2.1. Materials

Experimental fabrication of polyethylene sebacate resin has been reported [49]. Sodium tungstate dehydrates, ferrous ammonium sulfate hexahydrate, zinc chloride hexahydrate, and cetyltrimethylammonium bromide were obtained from ADWIC (Qalyubia, Egypt), Sigma-Aldrich (Saint Louis, MO, USA) and Merck (Kenilworth, NJ, USA).

### 2.2. Nano-Oxides Fabrication

Sodium tungstate and ferrous ammonium sulfate (mole ratio 2:1) were dissolved in DW individually and mixed under sturdy stirring. A dark-colored suspension appeared which was dissolved by the addition of oxalic acid [50]. Eventually, a transparent colored solution was obtained with a pH value of 1. The solution was shifted to a proper autoclaving system and aged for 2 days at 180 °C to get the final product. Further, zinc oxide was prepared through the coprecipitation process in presence of CTAB [51]. CTAB based zinc chloride hexahydrate was dissolved in distilled water (mole ratio of 0.8), 2 M aqueous NaOH medium was gradually added until pH raised to 11. Then the mixture was stirred for 2 h, shifted to autoclave, and aged for 2 days at 120 °C. After that centrifuged, washed, dried, and calcified for 4 h at 450 °C. The obtained nanocrystalline oxides are described as nWO_3_ and nZnO. The condensed porous crystalline phase was appeared with a particle size around 6–40 nm by using the Scherrer equation.

### 2.3. Nanocomposite Fabrication

**Figure 1:**
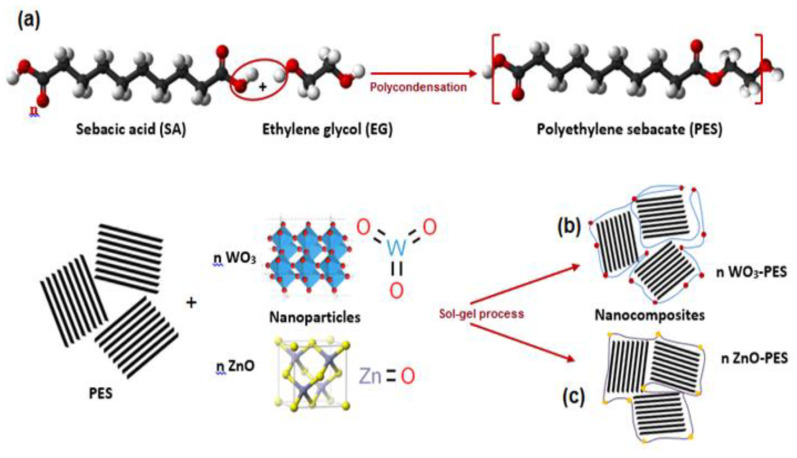
Sequence-based modeling of (**a**) PES, (**b**) PES-WO_3_ NC, and (**c**) PES-ZnO NC_._

Nanocomposites of WO_3_ and ZnO nanoparticles dispersed PES resin was fabricated throughout sequential proceedings as described in Figure 1. As an initial step, unbundling and dispersion of solid PES was operated by addition of about 10 g of resin to 50 mL of distilled water with sturdy stirring for 3 h at ambient temperature (Figure 1a). Thereafter, 0.05 g of nano-sized WO_3_ and ZnO as a solid powder was added quietly under vigorously stirring for 3 h (Wt.% 0.05 MO_x_/PES) [51]. Ultimately, the suspension was strongly stirred for 3h and aged for 48 h at 100 °C in a proper autoclaving system. Subsequently, the product was centrifuged, washed, and dried overnight. The yield was described as tungsten oxide and zinc oxide-based polyethylene sebacate nanocomposites (WO_3_-PES NC) and (ZnO-PES NC) (Figure 1b,c).

### 2.4. Characterization Techniques

Evaluation of weight throughout GPC analysis was performed by employing a Supremamax 3000 column at Polymer Standard Service with 2% acetic acid/0.2 M buffer sodium acetate as eluent (1 mL/min). FTIR technique was performed by employing an ATI Mattson model Genesis Series (Fremont, CA, USA), infrared spectrophotometer adopting KBr technique. XRD analysis was performed by employing a Philips Powder Diffractometer and monochromatized Cu Kα^1^ radiation in the range of 2θ = 4–80° at a potential of 40 KV and a current value of 40 mA with a running step of 2° in 2θ/min. SEM analysis was performed by employing a Jeol 5410 (Tokyo, Japan) instrument operated at a potential of 30 kV. TEM technique was performed by employing a Jeol instrument 2010 at a potential of 130 kV. The electrical activity was measured by using a Keithley test equipment-6517A Model-digit Electrometer (Solon, OH, USA). The microbial effect was examined by using the agar diffusion technique [52]. The tested composites were evaluated against Gram-positive bacteria (*Bacillus subtilis* ATCC 6633, *Staphylococcus aureus* ATCC 35556), Gram-negative bacteria (*Escherichia coli* ATCC 23282 and *Pseudomonas aeruginosa* ATCC 10145), Yeast (*Candida albicans* IMRU 3669), and Filamentous Fungus (*Aspergillus niger* ATCC 16404). The negative control was DMF showed no antimicrobial activity against the tested microorganisms, and the positive control was *Erythromycin* for bacteria and *Metronidazole* for yeast and fungus. All examinations were carried out in duplicates and the listed data are the average of the obtained results.

## 3. Results and Discussion

GPC values of the prepared PES were illustrated in detail in Table 1. Broadly, varied molecular weight measurements and determinations are detected owing to the step-growth melt polycondensation polymerization procedures. Under the extraordinary role of catalyst, a significant impact was exposed to molecular weight measurements of the polymer [53]. In the present work, the cured PES resin attained a total weight “M_n_” of 2103, which takes into account all the polymeric molecules that exist. The attained mode “the highest peak” molecular weight distribution “M_p_” value was 2422. Mass of the total polymeric chains “M_w_” value was 2621. As well, the catalytic action diminished the glycol overtaking and accordingly minimized the whole period of the reaction. The PES reveals the crucial mechanism and kinetics of the catalytic esterification process [54].

**Figure 2:**
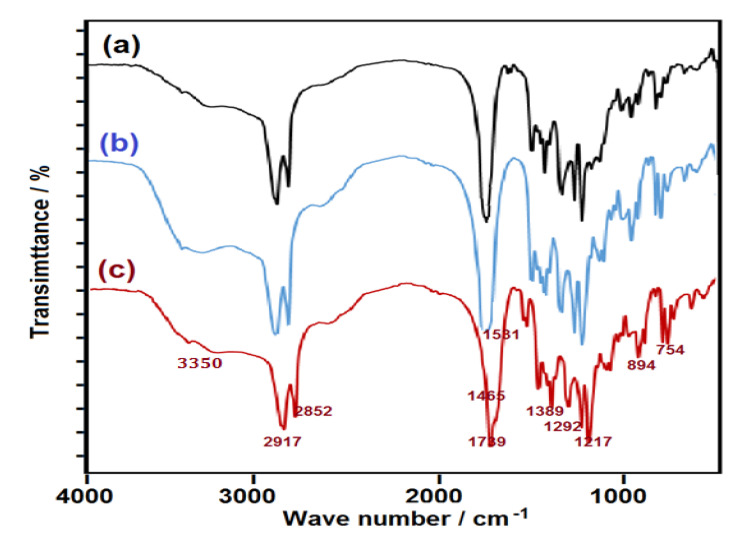
FTIR charts of (**a**) PES, (**b**) PES-WO_3_ NC, and (**c**) PES-ZnO NC composites_._

FTIR spectra of PES, WO_3_-PES NC, and ZnO-PES NC are shown in Figure 2. From the obtained spectra patterns, soft broad absorption peaks for OH groups manifested at 3350 cm^−1^. Two common symmetric stretching bands for CH– and CH= were revealed at 2852 and 2917 cm^−1^. Associated with ester chain formation, strong and sharp spectra were present at 1739 cm^−1^. Moderate spectra are shown at 1465 cm^−1^ due to the methylene group. Functional OH bending appeared as a medium absorption at 1389 cm^−1^. Two bands at 1292 and 1217 cm^−1^ were due to C–O of ester. C=C bending of CH= was exposed as two peaks at 894 and 754 cm^−1^. Owing to the low content of nano-sized oxides in PES, there are no considerable distinctions among the diagrams in which the spectrometric techniques are mainly employed to detect the hydrocarbon components [54].

**Figure 3:**
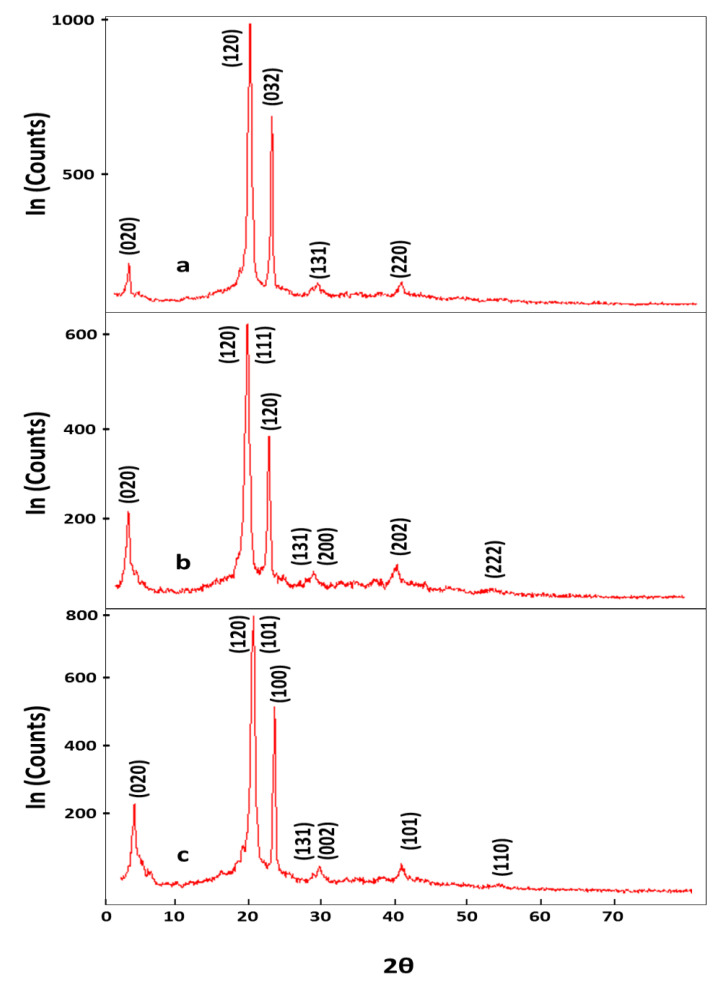
XRD charts of (**a**) PES, (**b**) PES-WO_3_ NC, and (**c**) PES-ZnO NC composites.

XRD plots (Figure 3) for PES, WO_3_-PES NC, and ZnO-PES NC reveal the presence of multiple diffraction patterns observed. The reflections of the crystalline structure were recorded in the 2θ range of 6–45°. The characteristic crystalline peaks appeared at around 2θ = 6.06° (30.4%), 21.75° (100%), and 24.64° (57.12%). The exposed value at 2θ = 21.75° is correlated to the high content of the ester series. Medium appearance at 2θ = 6.06° was due to rough form. The PES backbone contains prolonged polymer chains referring to various arrangements of polyester bindings [55]. ASTM 24-1148 and ASTM 89-0598 were reported in the diffraction plots of WO_3_ and ZnO. Otherwise, the characteristic strong and sharp diffraction peaks reveal the good crystallinity of the composites of PES-WO_3_ NC and PES-ZnO NC. The characteristic peaks at 2θ = 6.0°, 13.4°, 20.4°,21.7°, 24.5°, 29.8°, 30.6°, 38.8°, and 41.5° are related to the (020), (120), (032), (131), and (220) patterns of PES (JPDS: 04-0783). The high intensity and narrow peaks are detected at 2θ = 6.0°, 7.2°, 21.7°,21.9°, 24.6°, 24.7°, 30.6°, 38.8°,41.7°, and 45.4° are correlated to the (020), (020), (200), (120), (111), (120), (131), (200), (202), and (222) crystalline planes of the spherical phase of WO_3_ (JPDS: 43-0679). The strongest diffraction peaks observed at 2θ values of 5.9°, 8.0°, 11.4°, 17.3°, 20.2°, 21.6°, 24.5° 30.3°, 35.8°, 38.7°, and 41.2° corresponding to the planes (020), (120), (101), (100), (131), (002), (101), and (110) of ZnO (JPDS: 36-1451). The size of crystals was measured using the Scherrer equation to the peaks at 2θ = 24.64° and 2θ = 21.75° around 36 and 16nm.

**Figure 4:**
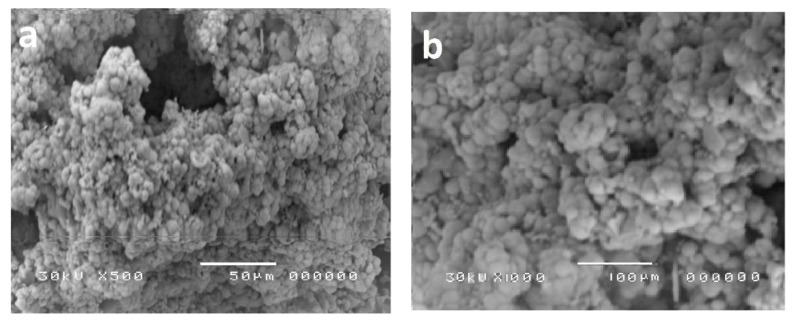
SEM graphs of the selected metal oxide nanoparticles (nMOx = (**a**)WO_3_/(**b**)ZnO)

**Figure 5:**
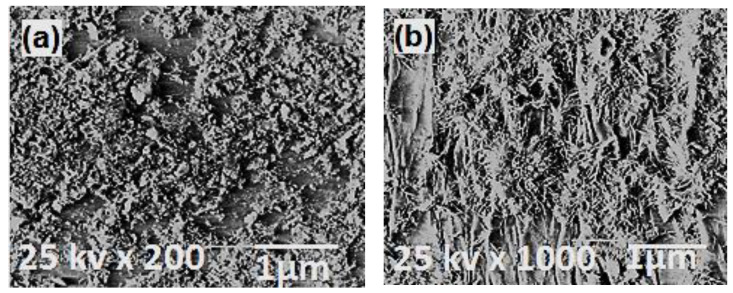
SEM graphs of (**a**) PES-WO_3_ and (**b**) PES-ZnO nanocomposites.

The surface morphology of metal oxide nanoparticles was characterized using the SEM technique. Before the discussion, metal oxides were found to be granulated particles. The agglomeration performance is mostly similar to all metal oxide particles prepared at relatively low temperatures [56]. With the elevating temperatures, the highly agglomerated crystalline domains were found to have fused spherical-shaped nanoparticles as shown in Figure 4. In the case of nanoparticles-based polymer (WO_3_-PES NC and ZnO-PES NC), SEM images (Figure 5) were found to have condensed porous shape for WO_3_-PES NC (Figure 5a) and diverged rode shape for ZnO-PES NC (Figure 5b).

**Figure 6:**
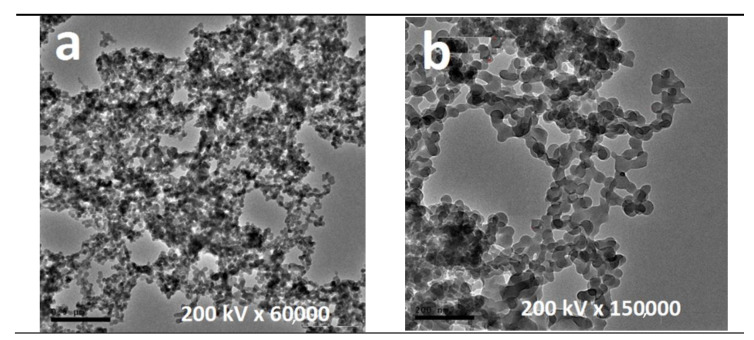
TEM graphs of the selected metal oxide nanoparticles (nMO_x_ = (**a**)WO_3_/(**b**)ZnO).

TEM micrographs of nano-sized oxides and polymer nanocomposites were studied. Regarding this issue, some micrographs of their polymeric structure and dispersed metallic particles are exhibited. As images display, pure nanoparticles were spherical flaked and randomly distributed in several areas, whereas particle agglomerates still existed and remained almost free areas (Figure 6).

**Figure 7:**
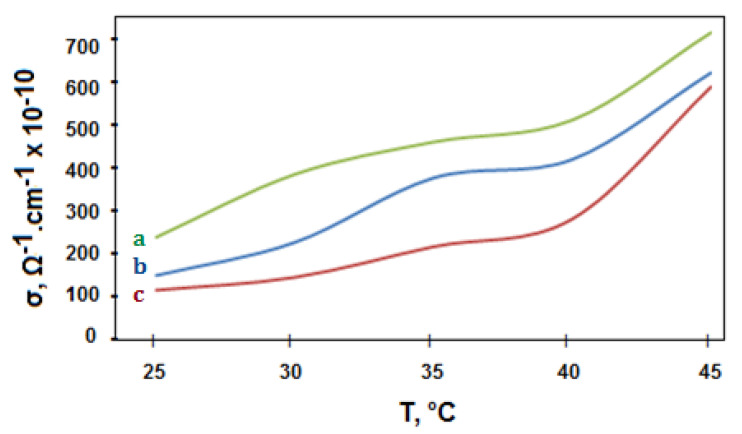
Conductivity diagram of (a) PES-ZnO, (b) PES, (c) PES-WO_3_ systems.

In general, the electrical conductivity of the polymeric composites can be promoted by adding a conductive nanomaterial into the polymer phase. The enhancement performance is owing to the formation of a permanent conductive network along with the polymer structure. The electrical conductivity values of polymer materials containing conductive nanofillers depend on nanophase content, dispersion area, temperature, and the particle size of the nanocomposites [57]. The electrical conductivity of PES, WO_3_-PES NC, and ZnO-PES NC is displayed in Figure 7. From the graphs, conductivity measurements progressively increased by increasing temperature. This action corresponds with the typical ionization performance of functional COOH in the polyester matrix. High conductivities are correlated to the nano solids and heat effects [58].

The microbial impacts of PES and its nanocomposites were evaluated against pathogenic Gram-negative bacteria, (*Pseudomonas aeruginosa ATCC 10145* and *Escherichia coli ATCC 23282*), Gram-positive bacteria (*Staphylococcus aureus ATCC 35556* and *Bacillus subtilis ATCC 6633*), and fungi (*Aspergillus niger ATCC 16404* and *Candida albicans IMRU 3669*) at a concentration of 5 mg/mL. The bacteria and yeast were grown on nutrient agar while the fungus was grown on Czapek’s Dox agar medium. Data in Table 2 indicate that the obtained composites’ antimicrobial effects range from moderate to slightly high on Gram-negative bacteria and from slightly high to high on Gram-positive bacteria and very high effect on fungi compared to the drug reference used. The values of the inhibition zones indicate the cytotoxic efficacy of these compounds against the studied fungi. The inhibition diameter values showed that the cytotoxic efficacies of these compounds are strongly related to their surface properties and the type of transition metal. The bactericidal effect of metal nanocomposite has been assigned to their small size and high surface to volume ratio, which allows them to interact closely with microbial membranes and is not solely owing to the release of metal ions in the medium. A cell wall exists around the outside of the microorganism cell membrane and it is essential to the survival of bacteria. Such a phenomenon can be explained based on Overtone’s concept and chelation theory [59,60,61].

## 4. Conclusions

The current study presents the preparation of WO_3_ and ZnO nanoparticles dispersed PES nanocomposites through the precipitation process and esterification reaction. Mass, structure, composition, morphology, conductivity, and microbial activity of the obtained systems were evaluated. Spectra of FTIR and XRD techniques confirmed the fabrication of polymer nanocomposites. It reveals the existence of several patterns for PES composites. The grain size of WO_3_ and ZnO were found in the nano-crystalline regime. SEM and TEM images show condensed spherical shaped nanomaterials embedded in the PES lattice. The nanocomposites based on nWO_3_ and nZnO exposed an electrical conductivity effect due to the interaction of ions in which the values are increased under heat. The antimicrobial results revealed a moderate effect on bacteria and a high effect on fungi for the nanocomposites based on inhibition zone. As a result, the current research contributes to the fabrication of reasonable nanomaterials to be under evaluation for their catalytic activity.

## Figures and Tables

**Table 1:** GPC of the prepared polyethylene sebacate (PES).

No.	Retention Time	Mn	Mw	MP (Daltons)	Mz (Daltons)	Mz + 1 (Daltons)	Poly- Dispersity
1	28.883	2103	2621	2422	3240	3888	1.246

**Table 2:** Antimicrobial activity of PES, PES-WO_3_, and PES-ZnO, measured by (mm).

	Test Organism	(+ve) Bacteria		(-ve) Bacteria		Fungi	
Compound ID		*Bacillus subtilis*	*Staph.* *aureus*	*Escherichia coli*	*Pseud.* *aeruginosa*	*Candida* *albicans*	*Aspergillus* *niger*
PES		13	18	12	12	28	25
PES-WO3		13	16	13	13	17	20
PES-ZnO		12	17	13	12	19	19
Reference		32	34	32	33	26	28

## Data Availability

Not applicable.
